# The value of ^18^F-PSMA-1007 PET/CT in identifying non-metastatic high-risk prostate cancer

**DOI:** 10.1186/s13550-020-00730-1

**Published:** 2020-11-10

**Authors:** Jun-jie Hong, Bo-le Liu, Zhi-qiang Wang, Kun Tang, Xiao-wei Ji, Wei-wei Yin, Jie Lin, Xiang-wu Zheng

**Affiliations:** Department of PET/CT, Radiology Imaging Center, The First Affiliated Hospital of Wenzhou Medical University, Xuefu North Rd, Wenzhou, 325000 Zhejiang People’s Republic of China

**Keywords:** ^18^F-PSMA-1007, Prostate cancer, High risk

## Abstract

**Background:**

Clinical management decisions on prostate cancer (PCa) are often based on a determination of risk. ^68^Ga-prostate-specific membrane antigen (PSMA)-11-positron emission tomography (PET)/computer tomography (CT) is an attractive modality to assess biochemical recurrence of PCa, detect metastatic disease and stage of primary PCa, making it a promising strategy for risk stratification. However, due to some limitation of ^68^Ga-PSMA-11 the development of alternative tracers is of high interest. In this study, we aimed to investigate the value of ^18^F-PSMA-1007 in identifying non-metastatic high-risk PCa.

**Methods:**

A total of 101 patients with primary non-metastatic PCa who underwent ^18^F-PSMA-1007 PET/CT were retrospectively analyzed. According to the European Association of Urology guidelines on PCa, patients were classified into intermediate-risk (IR) group or high-risk (HR) group. The maximum standardized uptake values (SUVmax) of the primary prostate tumor were measured on PET/CT images. The diagnostic performance of PET/CT for IR and HR PCa was calculated, and the relationship between the SUVmax of primary prostate tumor, prostate-specific antigen (PSA) level and Gleason score (GS) was analyzed.

**Results:**

Of all 101 patients, 49 patients were classified into IR group and 52 patients were classified into HR group. There was a significant positive correlation between PSA level/GS and SUVmax (*r* = 0.561, *r* = 0.496, *P* < 0.001, respectively). Tumors with GS 6 and 7a showed significantly lower ^18^F-PSMA-1007 uptake compared to patients with GS 8 and 9 (*P* < 0.01). SUVmax in patients of HR was significantly higher than those of IR (median SUVmax: 16.85 vs 7.80; *P* < 0.001). In receiver operating characteristic curve analysis, the optimal cutoff value of the SUVmax for identifying high-risk PCa was set as 9.05 (area under the curve: 0.829; sensitivity: 90.4%; specificity: 65.3%).

**Conclusion:**

^18^F-PSMA-1007 PET/CT showed the powerful diagnosis efficacy for high-risk PCa, which can be used as an objective imaging reference index for clinical reference.

## Background

PCa is one of the most common tumors in men worldwide [1]. Patients with those high-risk features (defined by the EAU guidelines on prostate cancer as T2c disease and/or sum Gleason score > 7 and/or serum PSA > 20 ng/ ml) predict a higher risk of metastasis, recurrence or death. The conventional method of identifying high-risk disease in the preliminary diagnosis fails to meet clinical needs. There is a need to develop new methods to allow for appropriate risk stratification for management, such as active surveillance programs, definitive therapy, prostatectomy, radiotherapy or up-front androgen deprivation. Incorporation of imaging to current primary PCa classifications for risk stratification can help achieve that unmet clinical need [2].

PSMA is a membrane-bound enzyme with high expression in prostate cancer cells and low expression in benign prostatic tissue [3]. Over the past few years, targeted imaging of PSMA has been used in various clinical managements, such as imaging-guided biopsy, staging of primary tumor, localization of biochemical relapse, planning of radiotherapy, prediction and assessment of tumor response to systemic therapy [4–7]. The PSMA expression level of PCa and tumor level, Gleason score and PSA stage before treatment have been proved definitely correlated, and the expression levels have been found to be a predictor for PCa progression [8–10]. PSMA-based PET/CT has also been reported to be enabling better tumor detection rate than standard radiologic imaging procedures [11].

Currently, ^68^Ga-PSMA-11 is a widely used tracer for PET imaging applications in the detection of PCa. Nevertheless, the disadvantage of ^68^Ga-PSMA PET/CT is that it has more bladder activity, as tracer accumulation in the urinary tract may influence the uptake evaluation of the prostate bed [12]. Recently, the new PSMA tracer, ^18^F-PSMA-1007, can eliminate this kind of disadvantage because of its hepatobiliary excretion owing to its moderate lipophilic characteristics. It has been used as a promising new PET tracer in the management of PCa [13, 14]. Furthermore, ^18^F-PSMA-1007 has longer half-life and higher physical spatial resolution than ^68^Ga-PSMA PET/CT, because ^18^F is cyclotron-produced with the larger activity amount [13]. In previous studies, ^18^F-PSMA-1007 has been reported that the intensity of tracer accumulation in the primary tumors of PCa patients correlated to GS and PSA level, and it is promising for accurate local staging of PCa [13, 15, 16]. Furthermore, it has similar or better diagnostic performance than ^68^Ga-PSMA-11 in local recurrence or metastasis [14, 17]. However, the major limitation of the studies is the relatively small number of patients, and there is limited published data on the diagnosis efficacy of ^18^F-PSMA-1007 PET/CT for high-risk PCa.

Thus, we intended to measure the intensity of tracer uptake in the primary prostate tumor and evaluate the value of ^18^F-PSMA-1007 PET/CT noninvasive imaging diagnostic strategies to identify the high-risk of PCa and tried to establish an objective imaging reference index.

## Materials and methods

### Patients

**Fig. 1:**
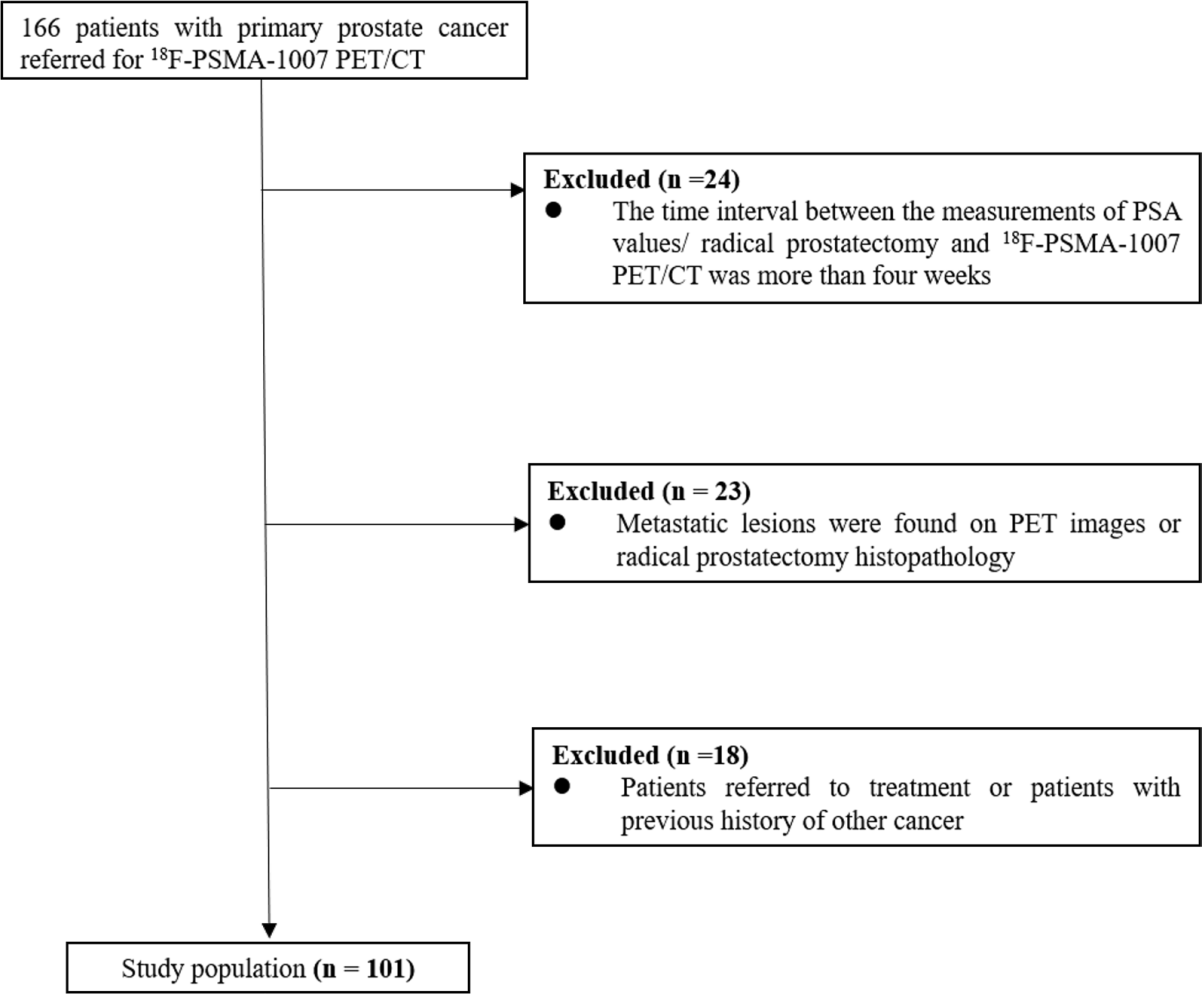
Flowchart of patient selection

In this retrospective study, we included the medical records of 101 PCa patients who underwent ^18^F-PSMA-1007 PET/CT imaging at our institution between March 2019 and August 2020. The inclusion criteria were: (1) all patients who underwent ^18^F-PSMA-1007 PET/CT imaging need complete clinical data; (2) all patients need to have radical prostatectomy (RP) histopathology. The exclusion criteria were: (1) the time interval between the measurements of PSA values/RP and ^18^F-PSMA-1007 PET/CT was more than 4 weeks (2) metastatic lesions were found on PET images or RP histopathology; (3) patients referred to treatment or patients with previous history of other cancer (Fig. 1). According to the EAU guidelines on prostate cancer [18], all patients were divided into Intermediate-risk (IR) group or high-risk (HR) group. The patients of the IR need to meet at least one of the following criteria: (1) PSA: 10–20 ng/ml; (2) Gleason score 7; (3) cT2b. As the same, the patients of the HR need to meet at least one of the following criteria: (1) PSA > 20 ng/ml; (2) Gleason score 8–10; (3) above cT2c. Due to the retrospective nature of the study, no formal approval from the ethics committee was required according to our national legislation.

### Radiopharmaceutical

^18^F-PSMA-1007 precursor, cassettes and reagents for the synthesis of ^18^F-PSMA-1007 were obtained from ABX advanced biochemical compounds (Radeberg, Germany). ^18^F-PSMA-1007 was prepared in a GE TracerLab FN synthesizer according to the one-step procedure described previously [19]. The radiochemical purity of the final product was > 90% as determined by high-performance liquid chromatography.

### Imaging protocol

^18^F-PSMA-1007 images were acquired from a body PET/CT scanner (Gemini 64 TF, Philips Medical Systems, Best, The Netherlands) and were performed approximately 120 min after IV injection of 4.0 MBq/kg ^18^F-PSMA-1007 (median activity: 291.2 MBq; range: 185.0–366.3 MBq). For attenuation correction, a low-dose unenhanced CT scan was performed from the skull base to the middle of the thigh, with the following parameters: tube voltage of 140 Kvp, tube current of 110 mA, detector collimation of 64 × 0.625 mm, pitch of 0.829, a tube rotation speed of 0.5 s, section thickness of 5 mm and reconstruction thickness of 2.5 mm, and was followed by the PET scan that matched the CT section thickness. A three-dimensional mode was used to obtain PET images with the following parameters: field of view, 576 mm; matrix of 144 × 144; slice thickness and interval, 5 mm. The emission scan time for each bed position was 1.5 min and the overlap between two adjacent bed positions was 50%.

### Image analysis

All ^18^F-PSMA PET/CT images were analyzed using a dedicated workstation (EBW3.0, Philips), which allowed the review of PET, CT and fused imaging data in axial, coronal and sagittal slices. PET imaging was interpreted independently by 2 experienced nuclear medicine physicians both of whom have more than 10 years of clinical experience and blind of all relevant clinical statistics. Any disagreement was resolved by consensus.

SUV_max_ of the primary tumors were acquired from the most intense uptake area in prostate gland. Areas in the whole body having uptake above the background activity were defined as metastatic. Typical pitfalls such as PSMA uptake in sacral and coeliac ganglia or in the stellate ganglia were frequently observed but were not considered pathological [20]. This interpretation criterion comes from the result of our clinical experience and consistent with published literature [21–24].

### Statistical analysis

Data analyses were performed with SPSS version 23.0 software (SPSS, Chicago, IL). Associations between GS, PSA value, and SUV_max_ of the primary tumor were described descriptively (nonparametric Spearman correlation coefficients). The differences between different subgroups were evaluated by using the Mann–Whitney U test and Kruskal–Wallis test. ROC curve analysis was used to determine the optimal cutoff value of the SUV_max_ for identifying high-risk PCa. For all statistical parameters, *P* values of less than 0.05 were considered statistically significant.

## Results

### Patients’ characteristics

The clinical characteristics of the enrolled 101 patients with GS 6–9 are summarized in Table 1. Among the 101 patients, the median age was 69 years (43–87 years). The proportions of patients enrolled in different subgroups were 51.5% and 48.5% for HR versus IR. All patients presented with a median PSA value of 11.113 ng/ml before the PET/CT scan (range: 0.970–178.200 ng/ml). The median SUVmax of all tumors was 11.6 (range: 4.3–77.7). (showed in Table 1).

**Table 1:** Patient characteristics

Patients (*n*)	101
Age median (range)	69 years (43–87)
PSA median (range)	11.113 ng/ml (0.970–178.200)
IR(*n*)	49
HR(*n*)	52
GS	
GS 6	4
GS 7a	30
GS 7b	37
GS 8	9
GS 9	21
Clinical T-stage	
T1c	41
T2a	9
T2b	7
T2c	39
T3a	2
T3b	3

*N*, number; IR, intermediate-risk group; HR, high-risk group; GS, Gleason score; GS 7a corresponds to GS 3 + 4; GS 7b corresponds to GS 4 + 3

### Correlation analysis

**Fig. 2:**
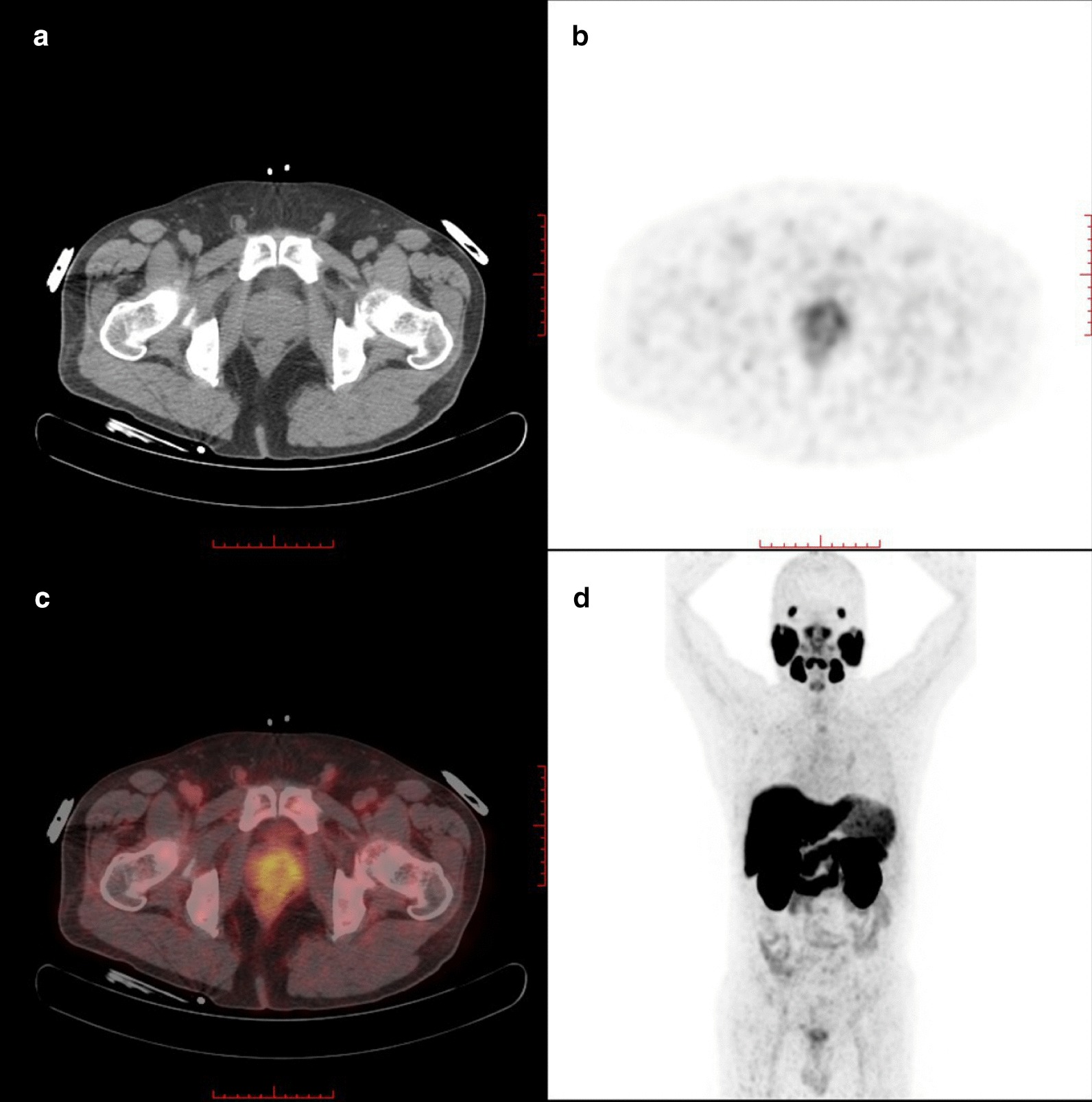
^18^F-PSMA-1007 PET/CT with CT (**a**), axial PET (**b**), and fused PET/CT (**c**) and maximum-intensity projection (**d**) images of a 56-year-old patient (GS, 7a; PSA, 6.780 ng/ml). This patient was classified into IR group. Axial PET (**b**) and fused PET/CT (**c**) images showed light scattered ^18^F-PSMA-1007 uptake in both sides of prostate gland (SUVmax: 5.20)

**Fig. 3:**
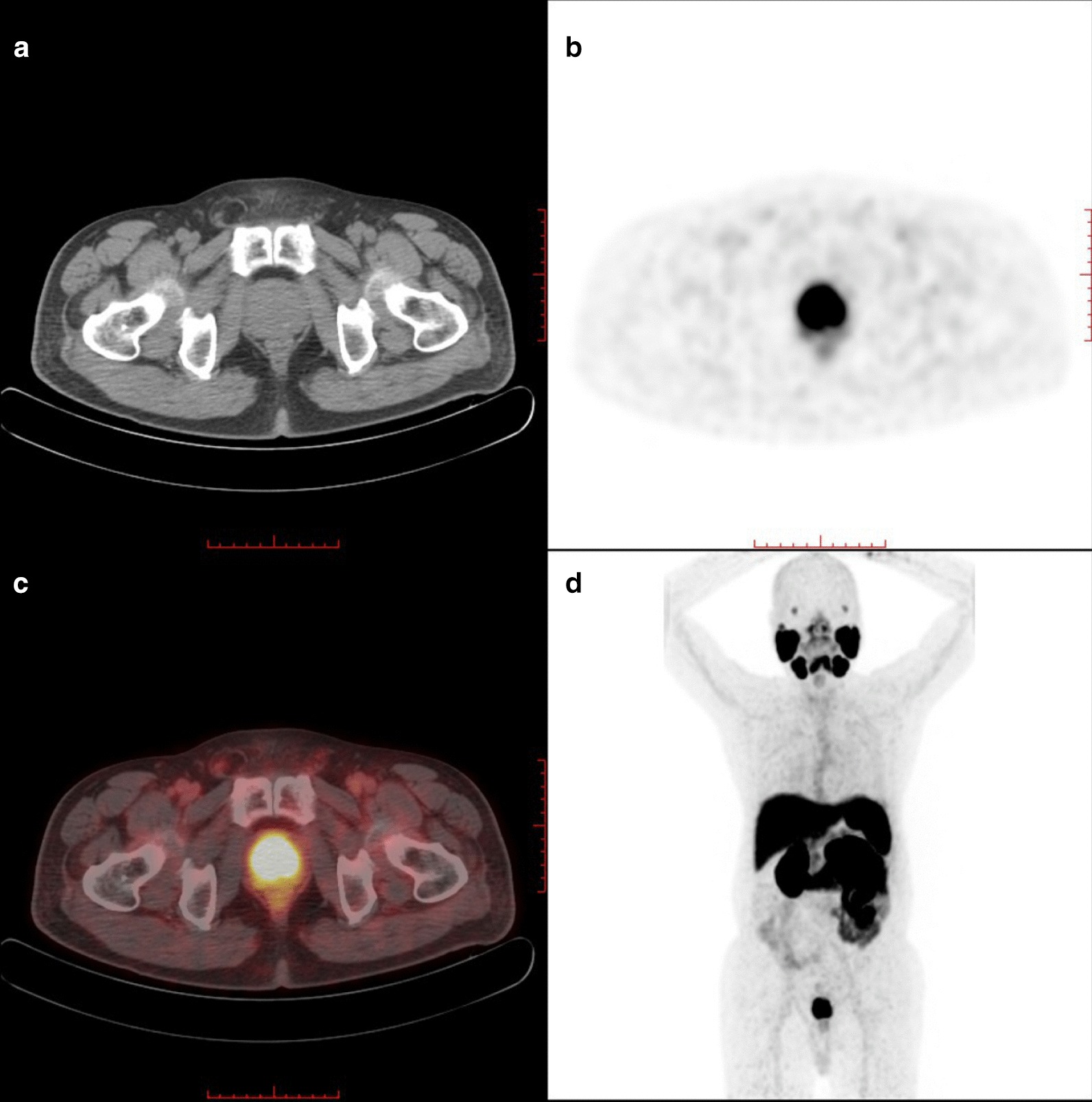
^18^F-PSMA-1007 PET/CT with CT (**a**), axial PET (**b**), fused PET/CT (**c**) and maximum-intensity projection (**d**) images of a 70-year-old patient (GS, 9; PSA, 37.910 ng/ml). This patient was classified into HR group. Axial PET (**b**) and fused PET/CT (**c**) images showed diffuse hypermetabolism in the prostate gland (SUVmax: 20.20)

**Fig. 4:**
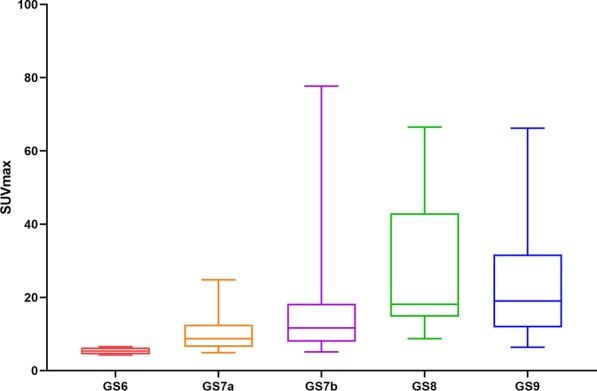
Comparison of ^18^F-PSMA-1007 uptake expressed in SUVmax value in primary tumors of different GS subgroups. Box plots demonstrate that higher GS exhibited statistically significant higher tracer uptake in the primary tumor

There was a statistically significant difference in median SUVmax between patients of HR and those of IR (16.85 vs 7.80, *P* < 0.001; Table 2). For the Gleason score, the detailed information about the SUVmax values of different GS subgroups was summarized in Table 3. Gleason score and SUVmax of primary tumors showed a significant positive correlation with each other (*r* = 0.496, *P* < 0.001). Combining GS and tumor-related tracer uptake, lower median SUVmax value was found in the subgroups GS 6 (SUVmax: 5.35) and GS 7a (SUVmax: 8.70) than in GS 7b (SUVmax: 11.60), GS 8 (SUVmax: 18.08) and GS 9 (SUVmax: 19.00). The result of Kruskal–Wallis test showed that the differences in SUVmax value between tumors with GS 6/7a and those with GS 8/9 were statistically significant (*P* < 0.01, respectively). Figures 2 and 3 show two examples for a GS 7a and a GS 9 PCa. A comparison of SUVmax for all GS subgroups is illustrated in Fig. 4. In terms of PSA level, there was a significant and strong positive correlation between the PSA value and the corresponding SUVmax value of the primary tumors (*r* = 0.561, *P* < 0.001).

**Table 2:** SUVmax value and PSA level of the primary tumor in different risk groups

	*N*	SUVmax, median (range)	SUVmax, mean ± SD	PSA, median (range)	PSA, mean ± SD
IR	49	7.80 (4.30–30.80)	10.03 ± 5.79	7.04 (0.970–18.190)	7.98 ± 3.604
HR	52	16.85 (6.40–77.70)	22.30 ± 15.97	21.81 (3.640–178.200)	25.95 ± 26.573

**Table 3:** SUVmax value of all primary prostate cancer in different Gleason score subgroups

	*N*	SUVmax, median (range)	SUVmax, mean ± SD
GS 6	4	5.35 (4.30–6.50)	5.38 ± 0.84
GS 7a	30	8.70 (4.90–24.80)	10.19 ± 5.28
GS 7b	37	11.60 (5.12–77.70)	15.72 ± 13.52
GS 8	25	18.08 (8.70–66.50)	27.99 ± 18.33
GS 9	21	19.00 (6.40–66.20)	23.35 ± 14.13
Total	101	11.60 (4.30–77.70)	16.34 ± 13.60

### ROC curve analysis

**Fig. 5:**
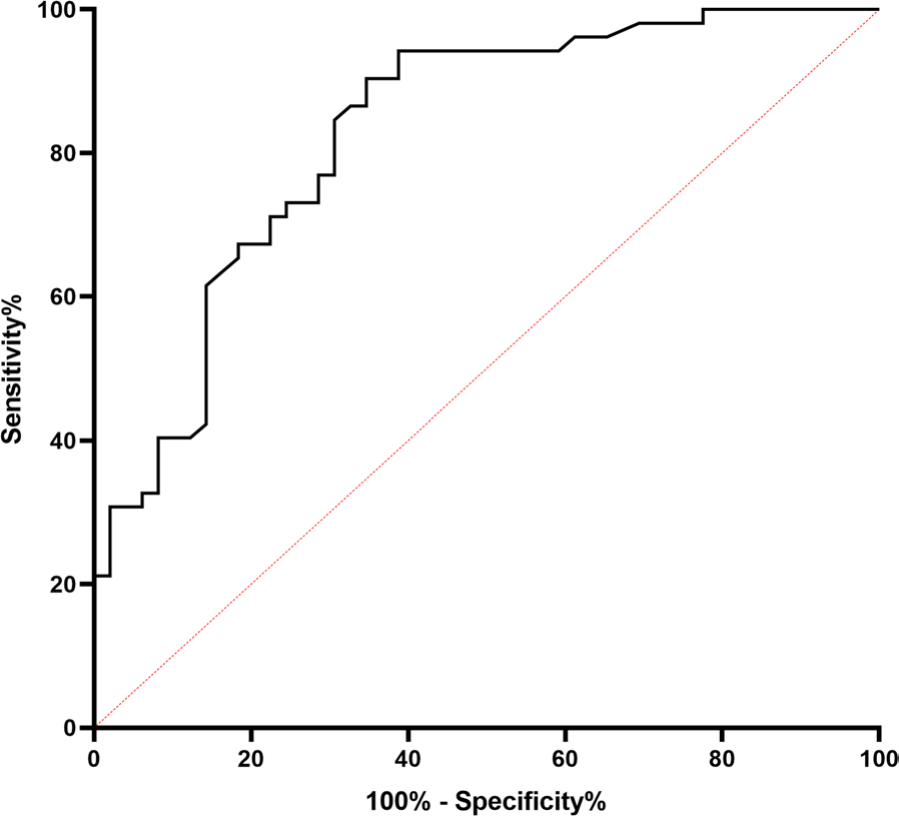
ROC curve of the SUVmax for high-risk prostate cancers. 95% confidence interval [CI], 0.749–0.909; sensitivity: 90.4%; specificity: 65.3%

Figure 5 showed the result of the ROC curve analysis for high-risk PCa. The AUC of the SUVmax was 0.829. The sensitivity, specificity, positive predicted value and negative predicted value were 90.4%, 65.3%, 73.4% and 91.4%, respectively. The optimal cutoff values of SUVmax was set as 9.05.

## Discussion

In this retrospective study, we found that there was a certain positive correlation between the intensity of ^18^F-PSMA-1007 accumulation and the GS/PSA level in the primary tumors of PCa patients. Furthermore, the SUV_max_ of the primary tumor was valuable for identifying high-risk PCa.

A timely and accurate diagnosis of high-risk PCa is front and center for the clinician. The commonly used risk classifications for the PCa are based on clinical stage, Gleason score by biopsy and PSA level before treatment. However, it is not absolutely reliable to evaluate the accuracy of GS in patients who have undergone 12-core random, transrectal ultrasound-guided (TRUS) biopsy. In the clinical work, it may also encounter the patients who refuse biopsy for a variety of reasons. Another problem with the scheme is the inherent inaccuracy in determining T stage [2]. Assessing disease by digital rectal examination has significant inter-observer variability. PSMA-PET/CT, as a noninvasive imaging diagnostic strategy, may compensate for these shortcomings. Recent studies found a statistically significant positive correlation between GS/PSA value and SUVmax of primary tumors on PSMA-PET/CT [13, 23]. Kesch et al. proved ^18^F-PSMA-1007 PET/CT and multiparametric magnetic resonance imaging had similar diagnostic performance in local staging of PCa [15]. In our study, the SUVmax showed a significant association with the presence of high-risk PCa. Patients of HR had significantly higher SUVmax than those of IR (*P* < 0.001). The AUC of the SUVmax of the primary tumor was 0.829, which can efficaciously identify non-metastatic high-risk patients with PCa. Therefore, we believe pathologists and clinicians may reduce missed diagnoses if they refer to PET images and results. Apart from that, PSMA-PET/CT may better screen out the patients of high risk, especially when the patients are unable to receive aspiration biopsy or the histology results of biopsy are not satisfactory.

The biological characteristics of PCa tissues vary greatly between different GS, which is an important indicator for the treatment and prognosis evaluation of PCa [18]. Thus, we also made the pairwise comparison between different GS subgroups and found that there were statistically significant differences in SUVmax between the subgroups of GS 6/7a and the subgroups of GS 8/9 (*P* < 0.01). There were no statistical differences in SUVmax value between tumors with 7b and those with GS 8/9, which was different from the result of previous studies on ^68^Ga-PSMA [23, 24]. Reasons for these discrepancies remain speculative. The patients of GS 8/9 account for a small proportion of all patients, which might be one of the reasons. Previous study had shown that compared with the subgroup GS 7b, the dangerous level of the subgroup GS 7a tumors could be treated conservatively without the need for a radical surgery [25]; thus, the distinguishment between the two subgroups was of great importance for clinical treatment. But it is worth noting that the SUVmax of primary tumor between these two subgroups has no statistical difference with a median SUVmax of 8.7 (GS 7a) and 11.6 (GS 7b), (*P* > 0.05). This finding was consistent with previous studies on ^68^Ga-PSMA [23, 24], which may reveal that the stage difference between GS 7a and GS 7b was not enough to cause a difference in SUVmax on PSMA-PET/CT.

The present study has some limitations that should not be neglected. Firstly, the retrospective nature of the analysis is the major limitation of our study, and further validation is required by multicenter studies with more patients. Secondly, the data of patients with GS 10 are lack in this study. Hence, the transferability of our data has yet to be assessed. Finally, we only focused on intra-prostatic ^18^F-PSMA-1007 uptake. The further researches about metastatic lesions or the impact of ^18^F-PSMA 1007 on the choice of treatment will be conducted in the further.

## Conclusion

In conclusion, ^18^F-PSMA-1007 was a great potential tracer for PCa PET/CT imaging. The intensity of tumor-related tracer uptake on ^18^F-PSMA-1007 PET/CT correlates with the PSA level and GS in primary PCa. Furthermore, ^18^F-PSMA-1007 PET/CT showed powerful diagnostic performance for risk stratification of primary PCa, which can be used as a reference index for identifying high-risk PCa.

## Abbreviations

PCa: Prostate cancer
EAU: European Association of Urology
PSA: Prostate specific antigen
GS: Gleason score
PSMA: Prostate specific membrane antigen
IR: Intermediate-risk
HR: High-risk
SUVmax: The maximum standardized uptake values
PET-CT: Positron emission tomography/computed tomography
TRUS: Transrectal ultrasound
RP: Radical prostatectomy
ROC: Receiver operating characteristic
AUC: Area under the curve

## Availability of data and materials

The datasets used and/or analyzed during the current study are available from the corresponding author on reasonable request.
